# Effects of stroke on changes in heart rate variability during hemodialysis

**DOI:** 10.1186/s12882-017-0502-0

**Published:** 2017-03-16

**Authors:** Jiun-Chi Huang, Chien-Fu Chen, Chia-Chu Chang, Szu-Chia Chen, Ming-Chia Hsieh, Yao-Peng Hsieh, Hung-Chun Chen

**Affiliations:** 1 Graduate Institute of Clinical Medicine, College of Medicine, Kaohsiung Medical University, Kaohsiung, Taiwan; 2 Division of Nephrology, Department of Internal Medicine, Kaohsiung Medical University Hospital, Kaohsiung Medical University, Kaohsiung, Taiwan; 3 Department of Internal Medicine, Kaohsiung Municipal Hsiao-Kang Hospital, Kaohsiung Medical University, Kaohsiung, Taiwan; 4 Faculty of Medicine, College of Medicine, Kaohsiung Medical University, Kaohsiung, Taiwan; 5 Division of Neurology, Department of Internal Medicine, Kaohsiung Medical University Hospital, Kaohsiung Medical University, Kaohsiung, Taiwan; 6 Division of Nephrology, Department of Internal Medicine, Changhua Christian Hospital, 135 Nanxiao Street, Changhua City, 500 Taiwan; 7 School of Medicine, Chung Shan Medical University, Taichung, Taiwan; 8 Program for Aging, China Medical University, Taichung, Taiwan; 9 Division of Endocrinology and Metabolism, Department of Internal Medicine, Changhua Christian Hospital, Changhua, Taiwan; 10 Graduate Institute of Integrated Medicine, China Medical University, Taichung, Taiwan; 11 Faculty of Renal Care, College of Medicine, Kaohsiung Medical University, Kaohsiung, Taiwan

**Keywords:** Autonomic nervous system, Heart rate variability, Hemodialysis, Stroke

## Abstract

**Background:**

Stroke and low heart rate variability (HRV) are both associated with an unfavorable prognosis in hemodialysis patients. The relationship between stroke and changes in HRV during hemodialysis remains unclear.

**Methods:**

This study measured differences between predialysis and postdialysis HRV (△HRV) in 182 maintenance hemodialysis patients, including 30 patients with stroke, to assess changes in HRV during hemodialysis, and also to compare results to 114 healthy controls.

**Results:**

All predialysis HRV measurements had no differences between stroke patients and those without stroke, but were lower than healthy controls. Postdialysis very low frequency (VLF) (*P* < 0.001), low frequency (LF) (*P* = 0.001), total power (TP) (*P* < 0.001) and the LF/high frequency (HF) ratio (*P* < 0.001) increased significantly relative to predialysis values in patients without stroke, whereas postdialysis HRV did not increase in stroke patients. After multivariate adjustment, dialysis vintage was negatively associated with △VLF (β = -0.698, *P* = 0.046), △LF (β = -0.931, *P* = 0.009), and △TP (β = -0.887, *P* = 0.012) in patients without stroke. Serum intact parathyroid hormone (β = -0.707, *P* = 0.019) was negatively associated with △LF. Total cholesterol (β = -0.008, *P* = 0.001) and high sensitivity C-reactive protein (β = -0.474, *P* = 0.012) were inversely correlated with the △LF/HF ratio in patients without stroke.

**Conclusion:**

HRV in hemodialysis patients is lower than in the general population. Increase in △HRV was observed in hemodialysis patients without stroke but not in stroke patients. This result suggests suppressed autonomic nervous reactions against volume unloading during hemodialysis, which might contribute to unfavorable outcomes in hemodialysis patients but even more so in those with prior stroke. Nephrologists should notice the importance of △HRV especially in high-risk patients.

## Background

End-stage renal disease (ESRD) has been recognized as a major public health problem worldwide. ESRD shares many traditional cardiovascular risk factors with cardiovascular disease (CVD), such as aging, hypertension, diabetes, and dyslipidemia [1]. CVD is the leading cause of death in patients with ESRD [2]. Among patients with CVD, the prevalence and incidence of stroke are drastically higher in dialysis patients than in the general population and patients with non-dialyzed chronic kidney disease (CKD), and are associated with an unfavorable prognosis [3–6]. The pathophysiological mechanisms mediating the poor outcomes of stroke in patients with ESRD remain unclear. However, these mechanisms appear to be related to an interaction between traditional and CKD-related nontraditional risk factors, as well as to certain characteristics unique to dialysis therapy, including uremic milieu, endothelial dysfunction, oxidative stress, drastic hemodynamic changes, and arterial stiffness [7]. Identifying high-risk individuals and developing risk stratification methods to initiate aggressive treatment interventions and improve outcomes for ESRD patients are clinically crucial.

Spectral analysis of heart rate variability (HRV) has been used as a noninvasive tool for accessing changes in the activity of the autonomic nervous system. HRV measurement provides prognostic and risk-stratification value for various populations, including patients undergoing chronic hemodialysis therapy [8–12]. Prior studies have shown that low baseline predialysis HRV is associated with adverse CVD outcomes and mortality in patients with ESRD [10–12]. However, change in HRV during hemodialysis appears to be a stronger predictor than predialysis HRV [13]. Although stroke is highly prevalent with unfavorable outcomes in hemodialysis patients, the relationship between stroke and the effects of hemodialysis on autonomic regulation measured by HRV remains unclear. In addition, the factors contributing to changes in HRV during hemodialysis are not well known. Therefore, the present study investigated the relationship between stroke and HRV, and to further explore the effects of hemodialysis on HRV in hemodialysis patients.

## Methods

### Ethics statement

The study protocol was approved by the Institutional Review Board of Kaohsiung Medical University Hospital. Informed consent was obtained in written form from patients and controls, and all clinical investigations were conducted according to the principles of the Declaration of Helsinki. All patients and controls gave consent for the publication of their information.

### Study patients and design

The study was conducted at the dialysis unit in a regional hospital in Taiwan. All patients in this hospital who were receiving routine hemodialysis treatment three times a week were included, excluding those receiving hemodialysis treatment at night. Patients with an implanted cardiac pacemaker or a history of atrial fibrillation were also excluded. Ultimately, 182 patients (81 male and 101 female) were enrolled in the study from May 2012 to July 2012.

To compare our HRV results for hemodialysis patients with the results for individuals without hemodialysis treatment, 114 controls (41 male and 73 female; mean age 54.5 ± 8.8 years) were recruited from the outpatient department of the hospital and general community. Among controls, 11 (9.6%) were with a history of diabetes, and 22 (19.3%) subjects were with a history of hypertension. The volunteer controls were all screened carefully to ensure they had no clinical history of stroke or on dialysis therapy, nor any manifestations of nervous system, cardiac, or pulmonary disease. The exclusion criteria included major forms of concurrent cardiac disease, such as atrial fibrillation, congestive heart failure, cardiomyopathy and previous myocardial infarction, as well as history of stroke, through a comprehensive interview and history taking, 12-lead electrocardiogram (ECG), and echocardiography.

### Hemodialysis treatment

All patients received conventional thrice-weekly hemodialysis treatment by using Toray 321 machines (Toray Medical Company, Tokyo, Japan). Each hemodialysis session was conducted for 3 to 4 h by using a dialyzer with blood flow rates ranging 250–300 mL/min and a dialysate flow rate of 500 mL/min.

### Definition of stroke

Stroke was defined as an episode of acute neurogenic dysfunction presumed to be caused by ischemia or hemorrhage, persisting for more than 24 h or until death [14]. The diagnosis of stroke was verified by patients’ brain imaging of computer tomography or magnetic resonance imaging and by evidence from the patients’ medical records.

### Electrocardiogram signal processing

Detailed procedures for the measurement of HRV have been reported previously [15–17]. In brief, a pericardial ECG was taken for 5 min before hemodialysis and after hemodialysis during the day (between 8 a.m. and 5 p.m.) with the patient lying quietly and breathing normally in a supine position for at least 10 min. ECG signals were recorded using an HRV analyzer (SS1C, Enjoy Research, Taipei, Taiwan) and an analog-to-digital converter with a sampling rate of 256 Hz. Digitized ECG signals were analyzed on-line and were simultaneously stored on a hard disk for off-line verification. Signal acquisition, storage, and processing were performed on an IBM-compatible portable personal computer. The computer algorithm identified each QRS complex and rejected each ventricular premature complex and noise according to its likelihood in a standard QRS template.

### HRV frequency domain analysis

Frequency domain analysis was performed using a non-parametric method of fast Fourier transformation (FFT). The direct current component was deleted, and a Hamming window was used to attenuate the leakage effect [18]. For each time segment (288 s; 2048 data points), our algorithm estimated the power spectrum density based on FFT. The resulting power spectrum was corrected for attenuation resulting from the sampling and the Hamming window. The power spectrum was subsequently converted into standard frequency domain measurements as defined previously [18], including very low frequency (VLF) (0.003–0.04 Hz), low frequency (LF) (0.04–0.15 Hz), and high frequency (HF) (0.15–0.40 Hz), as well as the LF/HF ratio. LF was normalized by the percentage of total power (TP) in order to determine the sympathetic influence on HRV. A similar procedure was applied to HF, which is mediated predominantly by vagal activity. In addition, TP of the spectrum of ≤ 0.40 Hz was calculated. The values calculated from each power spectrum, including VLF, LF, HF, TP, and the LF/HF ratio, were expressed in natural logarithmic form to obtain a normal distribution [15].

### Collection of demographic, medical, and laboratory data

Demographic and medical data including age, sex, and comorbid conditions were obtained from medical records and interviews with patients. The body mass index was calculated as the weight in kilograms divided by the square of height in meters. Venous blood was collected after an overnight fast for measuring various biomarkers by using an autoanalyzer (Roche Diagnostics GmbH, D-68298 Mannheim COBAS Integra 400). Serum intact parathyroid hormone (iPTH) concentration was evaluated using a commercially available two-sided immunoradiometric assay (CIS Bio International, France). Blood samples were obtained within 1 month of study enrollment. Kt/V was evaluated as a marker of dialysis efficiency and determined using the Gotch procedure [19]. Ultrafiltration percentage was calculated as the difference between predialysis and postdialysis weight divided by predialysis weight. Cardiothoracic ratio was measured on chest x-ray.

### Statistical analysis

Statistical analysis was performed using SPSS version 17.0 (SPSS Inc., Chicago, IL, USA) for Windows. Data are expressed as percentages, mean ± standard deviation, or mean ± standard error of the mean for HRV measurement values, or median (25^th^–75^th^ percentile) for dialysis vintage, triglycerides, high-sensitivity C-reactive protein (hs-CRP), and iPTH levels. The differences between groups were analyzed using the Chi-square test for categorical variables and using the independent *t*-test for continuous variables with approximately-normal distribution, or using the Mann–Whitney *U* test for continuous variables with skewed distribution. The paired *t*-test was used to compare HRV measurements before and after hemodialysis. We further examined whether stroke was associated with changes in HRV measurements by using an analysis of covariance (ANCOVA) after multivariate adjustment for age, sex, current smoking habits, diabetes, hypertension, coronary artery disease, ultrafiltration percentage, cardiothoracic ratio > 0.5, log-transformed hs-CRP, and use of angiotensin converting enzyme (ACE) inhibitors or angiotensin II receptor blockers (ARBs), beta-blockers, and calcium channel blockers. Changes in HRV measurements (△HRV) were defined as postdialysis HRV values minus predialysis HRV values. Multiple forward linear regression analysis was used to identify the factors associated with △HRV. A difference was considered significant if the *P* < 0.05.

## Results

A total of 182 maintenance hemodialysis patients and 114 controls were enrolled and analyzed in this study. The mean age of the hemodialysis patients and controls were 61.2 ± 11.3 years and 54.5 ± 8.8 years, respectively. Table 1 shows a comparison of HRV measurements between the hemodialysis patients and controls. Compared with those for the controls, the HRV measurements for hemodialysis patients were all significantly lower, except for the LF/HF ratio. In the linear regression analysis, after adjustment for age, sex, diabetes mellitus, hypertension, stroke and cardiovascular disease, hemodialysis had negative associations with all HRV parameters, except for the LF/HF ratio.

**Table 1:** Comparison of HRV parameters between hemodialysis patients and controls

Frequency domain HRV parameters	Hemodialysis patients (*n* = 182)	Controls (*n* = 114)	*P*-value
VLF (ln ms^2^)	4.1 ± 0.1	6.0 ± 0.1	<0.001
LF (ln ms^2^)	2.3 ± 0.3	5.1 ± 0.1	<0.001
HF (ln ms^2^)	2.2 ± 0.3	5.0 ± 0.1	<0.001
TP (ln ms^2^)	4.7 ± 0.1	6.7 ± 0.1	<0.001
LF/HF ratio [ln (ratio)]	0.08 ± 0.09	0.18 ± 0.07	0.405

*Abbreviations*: *HRV* heart rate variability, *VLF* very low frequency, *LF* low frequency, *HF* high frequency, *TP* total power

Among the 182 hemodialysis patients, thirty patients were verified with stroke (including 27 patients with prior ischemic stroke and 3 patients with prior hemorrhagic stroke). None of these patients were with acute state of stroke. Table 2 presents a comparison of baseline characteristics between hemodialysis patients with and without stroke. Compared with patients without stroke, stroke patients were more likely to have an older age, a shorter dialysis vintage, a higher prevalence of diabetes mellitus, hypertension, and coronary artery disease, a higher systolic blood pressure, lower serum albumin and creatinine levels, and a higher prevalence of cardiothoracic ratio > 0.5. All measurements of predialysis HRV parameters in stroke patients (VLF, LF, HF, TP, and the LF/HF ratio) were not significantly different from those in patients without stroke.

**Table 2:** Comparison of baseline characteristics between hemodialysis patients with and without stroke

Characteristics	All patients (*n* = 182)	Without stroke (*n* = 152)	With stroke (*n* = 30)	*P*-value
Age (years)	61.2 ± 11.3	60.4 ± 11.7	65.3 ± 8.4	0.009
Male gender (%)	44.5	45.4	40.0	0.689
Dialysis vintage (years)	5.9 (2.3–10.2)	6.6 (2.8–10.8)	2.8 (0.6–6.7)	0.001
Diabetes mellitus (%)	46.2	38.2	86.7	<0.001
Hypertension (%)	62.6	58.6	83.3	0.012
Coronary artery disease (%)	27.5	18.4	73.3	<0.001
Current smoking habits (%)	4.4	3.3	10.0	0.127
Systolic blood pressure (mmHg)	152.7 ± 26.5	150.2 ± 25.1	165.7 ± 30.2	0.004
Diastolic blood pressure (mmHg)	81.6 ± 14.3	81.8 ± 14.2	80.2 ± 14.8	0.573
Body mass index (kg/m^2^)	23.8 ± 3.4	23.9 ± 3.4	23.4 ± 3.6	0.520
Laboratory parameters				
Albumin (g/L)	38.4 ± 3.0	38.6 ± 2.9	37.2 ± 3.1	0.019
Fasting glucose (mg/dL)	119.2 ± 51.8	114.9 ± 43.0	142.0 ± 82.0	0.099
Triglycerides (mg/dL)^a^	131 (92–210)	127 (94–207)	153 (86–215)	0.314
Total cholesterol (mg/dL)^b^	182.5 ± 42.0	182.1 ± 41.8	184.6 ± 43.9	0.773
Hemoglobin (g/dL)	10.2 ± 1.1	10.3 ± 1.1	10.1 ± 1.0	0.443
Creatinine (mg/dL)	9.7 ± 2.1	9.9 ± 2.1	8.6 ± 1.7	0.004
Calcium (mg/dL)^c^	9.4 ± 1.0	9.4 ± 1.0	9.3 ± 1.3	0.707
Phosphorus (mg/dL)^d^	4.5 ± 1.0	4.4 ± 1.0	4.6 ± 1.1	0.384
iPTH (pg/mL)	351 (187–480)	351 (184–468)	348 (205–576)	0.710
hs-CRP (mg/L)	2.7 (1.0–6.5)	2.6 (1.0–6.6)	2.9 (0.8–6.3)	0.632
Kt/V (Gotch)	1.3 ± 0.2	1.3 ± 0.2	1.4 ± 0.2	0.736
Ultrafiltration percentage (%)	4.2 ± 1.4	4.2 ± 1.4	4.7 ± 1.6	0.060
Cardiothoracic ratio > 0.5 (%)	53.3	47.4	83.3	0.001
Medications				
ACE inhibitors or ARBs (%)	16.5	17.1	13.3	0.999
Beta-blockers (%)	17.6	16.4	23.3	0.180
Calcium channel blockers (%)	19.8	19.7	20.0	0.669
Frequency domain HRV parameters				
VLF (ln ms^2^)	4.1 ± 0.1	4.2 ± 0.1	3.6 ± 0.4	0.079
LF (ln ms^2^)	2.3 ± 0.3	2.6 ± 0.2	0.50 ± 1.27	0.107
HF (ln ms^2^)	2.2 ± 0.3	2.5 ± 0.2	0.51 ± 1.45	0.176
TP (ln ms^2^)	4.7 ± 0.1	4.8 ± 0.1	4.2 ± 0.4	0.054
LF/HF ratio [ln (ratio)]	0.08 ± 0.09	0.09 ± 0.10	-0.02 ± 0.27	0.652

*Abbreviations*: *iPTH* intact parathyroid hormone, *hs*-*CRP* high sensitivity C-reactive protein, *ACE* angiotensin converting enzyme, *ARB* angiotensin II receptor blocker, *HRV* heart rate variability, *VLF* very low frequency, *LF* low frequency, *HF* high frequency, *TP* total power

^a^For conversion into SI units (mmol/L): multiply with 0.0114

^b^For conversion into SI units (mmol/L): multiply with 0.0259

^c^For conversion into SI units (mmol/L): multiply with 0.25

^d^For conversion into SI units (mmol/L): multiply with 0.323

### Effects of hemodialysis on HRV

Table 3 shows the changes in HRV measurements after hemodialysis therapy. Compared with predialysis HRV values, postdialysis HRV, including VLF (*P* < 0.001), LF (*P* = 0.001), TP (*P* < 0.001) and the LF/HF ratio (*P* < 0.001), increased significantly after hemodialysis in patients without stroke. By contrast, no significant changes in postdialysis HRV measurements relative to the predialysis values were observed for stroke patients.

**Table 3:** Comparison of predialysis and postdialysis HRV parameters in hemodialysis patients with and without stroke

Frequency domain	Without stroke		With stroke	
HRV parameters	Predialysis	Postdialysis	Predialysis	Postdialysis
VLF (ln ms^2^)	4.2 ± 0.1	4.8 ± 0.1^†^	3.6 ± 0.4	2.8 ± 1.0
LF (ln ms^2^)	2.6 ± 0.2	3.4 ± 0.3^*^	0.50 ± 1.27	-0.17 ± 1.62
HF (ln ms^2^)	2.5 ± 0.2	2.9 ± 0.3	0.51 ± 1.44	0.69 ± 1.47
TP (ln ms^2^)	4.8 ± 0.1	5.5 ± 0.1^†^	4.2 ± 0.4	4.3 ± 0.4
LF/HF ratio [ln (ratio)]	0.09 ± 0.10	0.47 ± 0.08^†^	-0.02 ± 0.27	0.02 ± 0.27

^*^ *P*-value < 0.05 compared with predialysis HRV

^†^ *P*-value < 0.001 compared with predialysis HRV

Abbreviations are the same as in Table 1

We further performed ANCOVA to examine the main effect of stroke on HRV after multivariate adjustment for age, sex, current smoking habits, diabetes, hypertension, coronary artery disease, ultrafiltration percentage, cardiothoracic ratio > 0.5, log-transformed hs-CRP, and use of ACE inhibitors or ARBs, beta-blockers, and calcium channel blockers. Our analysis revealed that hemodialysis in patients without stroke were independently associated with an increase in postdialysis HRV, including VLF (*P* = 0.010), LF (*P* = 0.001), and TP (*P* = 0.048). No such association was observed for the LF/HF ratio (Table 4).

**Table 4:** Multivariate adjustment for main effects of stroke on △HRV parameters in hemodialysis patients

△HRV parameters	Parameter estimate (95% CI)	*P*-value
△VLF (ln ms^2^)	1.917 (0.463, 3.370)	0.010
△LF (ln ms^2^)	3.246 (1.291, 5.322)	0.001
△TP (ln ms^2^)	0.790 (0.009, 1.572)	0.048
△LF/HF ratio	0.513 (-0.140, 1.165)	0.122

Values expressed as parameter estimates and 95% confidence interval (CI)

Covariates in the multivariate model included age, sex, current smoking habits, a history of diabetes, hypertension, stroke, and coronary artery disease, ultrafiltration percentage, cardiothoracic ratio > 0.5, log-transformed hs-CRP and use of ACE inhibitors or ARBs, beta-blockers, and calcium channel blockers

### Determinants of △HRV in patients without stroke

Table 5 shows the unstandardized coefficient β of △HRV values, which were significant in patients without stroke, after adjustment for age, sex, dialysis vintage, current smoking habits, a history of diabetes, hypertension, and coronary artery disease, systolic and diastolic blood pressure, body mass index, levels of albumin, fasting glucose, triglycerides, total cholesterol, hemoglobin, creatinine, total calcium, phosphorus, iPTH, log-transformed hs-CRP, Kt/V, ultrafiltration percentage, cardiothoracic ratio > 0.5, and use of ACE inhibitors or ARBs, beta-blockers, and calcium channel blockers.

**Table 5:** Determinants of △HRV parameters of hemodialysis patients without stroke

△HRV parameters	Multivariate (Forward)	
	Unstandardized coefficient β (95% CI)	*P*-value
△VLF		
Dialysis vintage (log per 1 year)	-0.698 (-1.383,-0.013)	0.046
△LF		
Dialysis vintage (log per 1 year)	-0.931 (-1.622, -0.240)	0.009
iPTH (log per 1 pg/mL)	-0.707 (-1.298, -0.117)	0.019
△TP		
Dialysis vintage (log per 1 year)	-0.887 (-1.571, -0.202)	0.012
△LF/HF ratio		
Total cholesterol (per 1 mg/dL)	-0.008 (-0.013, -0.003)	0.001
hs-CRP (log per 1 mg/L)	-0.474 (-0.844, -0.105)	0.012

Values expressed as unstandardized coefficient β and 95% confidence interval (CI)

Covariates in the multivariate model included age, sex, dialysis vintage, current smoking habits, a history of diabetes, hypertension, and coronary artery disease, systolic and diastolic blood pressure, body mass index, albumin, fasting glucose, triglycerides, total cholesterol, hemoglobin, creatinine, total calcium, phosphorus, iPTH, log-transformed hs-CRP, Kt/V, ultrafiltration percentage, cardiothoracic ratio > 0.5, use of ACE inhibitors or ARBs, beta-blockers, and calcium channel blockers

In the multivariate forward analysis, a longer dialysis vintage was negatively associated with △VLF (unstandardized coefficient β = -0.698, *P* = 0.046), △LF (unstandardized coefficient β = -0.931, *P* = 0.009), and △TP (unstandardized coefficient β = -0.887, *P* = 0.012). A lower iPTH level was correlated with △LF (unstandardized coefficient β = -0.707, *P* = 0.019). Furthermore, the total cholesterol level (unstandardized coefficient β = -0.008, *P* = 0.001) and hs-CRP level (unstandardized coefficient β = -0.474, *P* = 0.012) were inversely correlated with the △LF/HF ratio in patients without stroke.

## Discussion

In the present study, we investigated the association between stroke and HRV in hemodialysis patients. All predialysis HRV measurements in stroke patients were not significantly different from those in patients without stroke, but were lower than in the controls. Compared with predialysis HRV values, all postdialysis HRV measurements, except for HF, significantly increased in patients without stroke, whereas no increase in postdialysis HRV was observed in stroke patients. Dialysis vintage, serum iPTH, total cholesterol, and hs-CRP levels contributed to changes in △HRV in patients without stroke.

The baseline HRV values were lower in hemodialysis patients compared with healthy controls in our study, and this finding was in line with the report by Vita et al [20]. Although the actual underlying mechanisms remain uncertain, activation of the renal afferents, structural remodeling of the heart and vasculature, as well as impaired reflex control of autonomic activity may play pivotal roles in ESRD patients [21].

HRV has been reported to be suppressed in both hemorrhagic and ischemic stroke patients [22–24]. Korpelainen et al. [23] demonstrated that, compared with control subjects, patients had impaired VLF and LF in the acute phase of ischemic stroke at hemisphere, as well as in the late state of stroke. Previous studies have demonstrated that baroreflex impairment occurred in ischemic and hemorrhagic stroke [25, 26]. Impaired baroreflex function might lead to altered cardiovascular regulation in stroke. Studies of ESRD patients on dialysis therapy have also shown evidence of decreased HRV [20, 27]. Renal failure and stroke have both been associated with impaired autonomic function. However, the relationship between stroke and HRV in ESRD patients has not previously been elucidated. In the present study, no significant differences in predialysis HRV values between hemodialysis patients with and without stroke were observed. Our proposed reason for this observation is that HRV in hemodialysis patients is affected by factors other than stroke, such as the composition and temperature of the dialysate [28, 29], and fluid overload levels [30].

Changes in HRV during hemodialysis treatment have been demonstrated in patients with ESRD [31, 32]. Emerging evidence indicates that the ability of changes in △HRV is a better predicting factor for unfavorable outcomes in comparison of predialysis HRV [13]. Although patients with or without stroke and hemodialysis did not significantly differ in predialysis HRV, an increase in △HRV was observed in patients without stroke but not in patients with stroke. Our HRV results further elucidate the relationship between stroke and the effects of hemodialysis on autonomic nervous system regulation. In patients without stroke, postdialysis values of VLF, LF, TP, and the LF/HF ratio increased significantly relative to the predialysis values, suggesting a shift toward sympathetic predominance of sympatho-vagal balance by volume unloading during hemodialysis. These findings are comparable with a study by Tong and Hou [32]. By contrast, there were no significant differences between predialysis and postdialysis HRV values in stroke patients. This observation implies an autonomic cardiovascular dysregulation and impaired sympathetic reaction against fluid removal during hemodialysis treatment in stroke patients. The pathophysiological mechanisms of such dysfunction remain unclear. Accumulating evidence suggests that the central autonomic network, especially the insular cortex, seems to play a key role in modulating the baroreflex function [33]. Furthermore, baroreflex impairment has been independently associated with unfavorable outcomes after stroke [26, 34].

The process of ultrafiltration during hemodialysis reduces circulating volume and arterial pressure, which could be sensed by baroreceptors and trigger cardiac sympathetic response. In the present study, we found that a longer dialysis vintage was inversely associated with increases in postdialysis VLF, LF, and TP, relative to the predialysis values in hemodialysis patients without stroke. It suggests that the effect of fluid removal on HRV might be blunted as dialysis vintage becomes longer. Chrapko et al. [35] evaluated the cardiac sympathetic nervous system function by using iodine-123 meta-iodo-benzylguanidine (^123^I-mIBG) myocardial uptake in 36 hemodialysis patients. Their results showed that patients with longer duration of hemodialysis had impaired function of the cardiac sympathetic system, thus supporting our hypothesis. The cardiac sympathetic response might deteriorate with increased duration of hemodialysis. This phenomenon is also similar to the work by Tamura et al. [36], which demonstrated that a longer duration of hemodialysis was associated with decreased HRV.

Secondary hyperparathyroidism is common in hemodialysis patients and may play a role in dysfunction of the autonomic nerve system. Polak et al. [37] reported low LF and HF in 40 hemodialysis patients with high serum levels of iPTH, indicating deterioration in autonomic activity. Increase in iPTH may cause endothelial dysfunction and vascular calcification, leading to enhanced coronary risk. Furthermore, Zhang et al. [38] found that serum level of iPTH was significantly correlated with decreased HRV, and parathyroidectomy might reverse the cardiovascular risk in CKD stage 5 patients. Consistent with previous studies, we found that higher iPTH levels were negatively associated with increased postdialysis LF in patients without stroke. Controlling iPTH levels might help to increase postdialysis HRV in such patients.

In addition, our results show that hypercholesterolemia was negatively associated with the △LF/HF ratio in patients without stroke. Several studies have examined the relationship between dyslipidemia and HRV in the general or ESRD population. For example, Christensen et al. [39] reported an association between hypercholesterolemia and low HRV. The association between dyslipidemia and low HRV is not fully understood. In both animal and human studies, the autonomic nervous system has been reported to participate in the regulation of cholesterol synthesis [40, 41]. Shanygina et al. [41] showed that vagotomy was associated with increased levels of total and low-density lipoprotein cholesterol in an animal model. Hence, impaired sympatho-vagal balance might be associated with dyslipidemia. Our findings for hemodialysis patients without stroke are in line with those of prior research. Treating dyslipidemia in such patients might have a potential role of improving HRV. Moreover, we found that hs-CRP level was inversely correlated with the △LF/HF ratio in patients without stroke. Autonomic dysregulation might be worse in patients with inflammation. This finding is comparable with the report by Chandra et al. [42] in CKD stages 3–5 patients. As inflammation is an important risk factor for unfavorable outcomes, therapeutic interventions targeting inflammation need to be developed in clinical trials. Further study is warranted to understand the pathways responsible for these observed associations.

There were several limitations in the current study. First, the study was a cross-sectional design with inherent weaknesses including unclear causal relationships and a lack of long-term observation of outcomes. Subjects of controls should be sex, age, and comorbidity*-*matched to compare with study patients. Second, a 24-h Holter ECG for HRV measurements would provide substantially more data. A previous report, however, indicated that 5 min is sufficient for short-term HRV analysis [43]. Third, the number of study patients was relatively small. The FFT method for HRV analysis might not be perfectly accurate, and using a robust period detection may have better performance [44]. Moreover, participants in this study are survivors of stroke, and thus at risk for survivor bias. These results might not be generalizable to all hemodialysis patients who experienced stroke. Changes in serum glucose during hemodialysis, markers of fluid overload as well as cardiac function may affect HRV, and should be considered in the further studies. A future large-scale prospective investigation including non-linear parameters of HRV and assessing baroreflex using heart rate and blood pressure variability is necessary.

## Conclusions

Baseline predialysis HRV did not significantly differ between hemodialysis patients with stroke and those without stroke, but was lower than controls. An increase in △HRV was demonstrated in patients without stroke but was not observed in stroke patients after hemodialysis. The results suggest a suppressed autonomic nervous reaction against volume unloading during hemodialysis, which might be a plausible contributing factor of unfavorable outcomes in stroke patients. Long dialysis vintage, high iPTH levels, high total cholesterol, and high hs-CRP levels were associated with a lesser degree of HRV increase after hemodialysis in patients without stroke. As the ability of increase in △HRV is a novel predictor of outcomes in hemodialysis patients, physicians and nephrologists should not only notice the importance of predialysis HRV, but also the changes in postdialysis HRV in high-risk patients.

## Abbreviations

ACE: Angiotensin converting enzyme
ANCOVA: Analysis of covariance
ARB: Angiotensin II receptor blocker
CKD: Chronic kidney disease
CVD: Cardiovascular disease
ECG: Electrocardiogram
ESRD: End-stage renal disease
FFT: Fast Fourier transformation
HF: High frequency
HRV: Heart rate variability
hs-CRP: High sensitivity C-reactive protein
iPTH: Intact parathyroid hormone
LF: Low frequency
TP: Total power
VLF: Very low frequency
